# Mechanical and Combustion Properties of Agglomerates of Wood of Popular Eastern European Species

**DOI:** 10.3390/ma14112728

**Published:** 2021-05-21

**Authors:** Marek Molenda, Józef Horabik, Piotr Parafiniuk, Anna Oniszczuk, Maciej Bańda, Justyna Wajs, Ewa Gondek, Marcin Chutkowski, Aleksander Lisowski, Joanna Wiącek, Mateusz Stasiak

**Affiliations:** 1 Institute of Agrophysics, Polish Academy of Sciences, Doświadczalna Str. 4, 20-290 Lublin, Poland; m.molenda@ipan.lublin.pl (M.M.); j.horabik@ipan.lublin.pl (J.H.); p.parafiniuk@ipan.lublin.pl (P.P.); m.banda@ipan.lublin.pl (M.B.); j.wajs@ipan.lublin.pl (J.W.); j.wiacek@ipan.lublin.pl (J.W.); 2 Department of Inorganic Chemistry, Medical University of Lublin, Chodźki 4a, 20-093 Lublin, Poland; anna.oniszczuk@umlub.pl; 3 Department of Food Engineering and Process Management, Warsaw University of Life Sciences, Nowoursynowska 159C, 02-787 Warsaw, Poland; ewa_gondek@sggw.pl; 4 Department of Chemical and Process Engineering, Faculty of Chemistry, Rzeszow University of Technology, Powstańców Warszawy 6, 35-959 Rzeszow, Poland; ichmch@prz.edu.pl; 5 Department of Agricultural and Forest Engineering, Faculty of Production Engineering, Warsaw University of Life Sciences, Nowoursynowska 166, 02-787 Warsaw, Poland; aleksander_lisowski@sggw.pl

**Keywords:** sawdust, pellet properties, wood, combustion, drop resistance, quality

## Abstract

The objective of the reported project was to produce wood agglomerates from popular East European species to determine their strength and combustion properties. Closed-die pellets were produced from sawdust of six types of wood common on the East European market: pine, willow, oak, poplar, birch, and beech. The properties of pellets, determined by the type of wood, were influenced by the compaction pressure and the moisture content of the sawdust. The highest average pellet density was obtained for oak sawdust, while the lowest density was obtained for poplar pellets. Expansion of pellets after removing from the die was found to be dependent on the wood species, and as expected, on compaction pressure. The pellet expansion increased after 2 h of conditioning in the laboratory and with an increase in moisture content. The highest and the smallest strength were obtained for oak pellets and for birch sawdust, respectively. The strength of the pellets increased by more than 100% with an increase in the compaction pressure from 60 MPa to 120 MPa. The average strength decreased by 65% with increasing moisture content. For all tested materials, drop resistance remained at a high level, acceptable in industrial practice. The highest calorific value of 18.97 MJ/kg was obtained for pine pellets. The highest ash value of 1.52% was obtained for willow pellets and the lowest value of 0.32% for pine pellets.

## 1. Introduction

The contribution of green energy to the total energy balance is constantly increasing. For this purpose, energy plants are increasingly being powered by agricultural, forestry, and plant products. A typical preliminary treatment of any waste biomass from the wood and agriculture industry comprises a densification of this biomass to create a homogeneous compacted material, making it convenient for dosing and transport [1,2,3,4,5,6,7,8,9]. Generally, the material is compacted into briquettes and pellets. In recent years, wood pellets have become the main source of co-firing in European power plants [3]. The materials often comprise mixtures with different proportions of materials from various origins [10]. Pelletization allows better material feeding, with less dust formation [5].

Over the last few years, to meet the increasing demand the importation of pellets from outside Europe has continued to increase. The transport of biofuel pellets is profitable for economic and environmental reasons, even if milling is necessary before combustion in pulverised feed systems [11]. The increasing use of pellets also raises the need to study their properties, including any properties that change during storage. Graham et al. [1] studied the mechanical degradation of white wood pellets in indoor and outdoor stockpile storage over a month-long period, and determined the optimal conditions for pellet storage. Other studies on the shelf lives of stored pellets have been conducted in many laboratories worldwide. Graham et al. [12] found that indoor storage caused a slight decrease in pellet quality (as obtained from a wood shelf life) of approximately 3% after 20 months. Untreated white wood, steam-exploded wood, and torrified wood were considered. Outdoor storage led to a much higher level of mechanical degradation. Steam-exploded pellets had better mechanical durability.

The storage and handling properties of pellets obtained with binders have also been widely studied [2,6,13]. Pellets obtained from torrified biomass are also of interest to researchers [2]. In addition, laboratory-scale research has been extended to industrial-scale research. The main objective of Dafnomilis et al. [3] was to assess the state-of-the-art wood pellet handling conducted in import terminals. Miranda et al. [4] presented the properties of pellets produced from different alternative sources. The properties suitable for pellet production were analysed for agricultural activities, biomass forestry, and other related industries. Filbakk et al. [14] analysed the effects of the contributions of different woods mixed with bark on the mechanical properties of pellets. Gilbert et al. [5] studied the effects of pelletisation conditions on the product properties of switch grass and wheat straw, using a simple compression rig. Niedziółka et al. [10], Stasiak et al. [13], and Adapa et al. [15] conducted studies on the mechanical properties of pellets obtained from agricultural biomasses. Li and Liu [8] analysed the high-pressure binderless compaction of wood processing residues and other biomass waste materials (such as hardwood, softwood, and bark) in the forms of sawdust mulches and chips. The authors used a punch-and-die process at room temperature, and pressures from 30 to 138 MPa. They found that the compaction of mulches into dense and strong logs was the easiest. The process was more difficult for sawdust, and the most difficult for chips. Li and Liu found that the initial moisture content of a material should range from 5% to 12% for the production of good quality agglomerates. They indicated that a material moisture content of 8% was the best for this purpose. These authors also determined the influences of the moisture, compaction pressure, compaction speed pressure holding time, and particle size and shape on the agglomeration process. The mechanical and combustion behaviours of pellets made from different types of biomass, their blends, and blends with coal were studied by Gil et al. [16]. The authors determined an abrasion index, and conducted a thermogravimetric analysis to select the pellets with the highest durability. The authors reported that biomass blends presented combustion profiles similar to those obtained for the individual raw materials, and that the addition of coal to the blends did not influence the properties of the pellets. Krizan et al. [17] analysed the relationships between the technological and material variables of beech sawdust during its densification. The mutual interactions between the compression pressure, temperature, and particle size were determined.

A review of the literature has shown that so far, no analysis has been conducted to examine the differences in the mechanical properties, combustion properties, and durability of pellets made from various types of wood. The type of raw material used for the production of pellets or briquettes is of great importance for matching the final product and providing appropriate energy quality. Although there is a significant amount of literature on the properties of pellets and briquettes produced from different materials or mixtures of these materials, no literature has been found that addresses how to differentiate and analyse the properties of pellets depending on the type of wood. Such an approach is necessary, as wood waste on the market is not produced from a single tree species.

Therefore, the aim of the present study was to analyze the mechanical and combustion properties of pellets obtained from various wood species popular in Eastern European countries. The quality of pellets is related to their physical, chemical and mechanical properties. The parameters examined in this study are among the most important recommended in existing standards for the characterization of pellets. Sawdust waste is a raw material used for obtaining pellets in the domestic market. Accordingly, the mechanical and combustion properties of pellets produced from sawdusts from different types of wood were determined. The quality levels of agglomerates produced without binders and additives were also examined. The findings presented in this project provide valuable knowledge for technologists and users of these materials. The pellets used in this study were produced in a single-matrix pelletiser [6,15,18,19], higher-performance laboratory pelletiser, and semi-industrial pelletiser [20,21,22,23,24]. In the project presented herein, an unheated matrix was used.

## 2. Materials and Methods

The material used for the production of pellets was sawdust obtaining from sawing six types of wood with a standard circular saw. The sawdust was created by cutting boards. The material was bark-free. The pieces of wood were dried and conditioned under laboratory conditions for half of a year. The pieces of wood used for cutting were obtained from six types of wood popular in the furniture industry: pine, birch, oak, poplar, willow, and beech. The size distribution was measured by the weight method using a sit-stack and laboratory shaker. A sieve analysis was performed on a vibrating heap of sieves, with mesh sizes as follows: 0.1, 0.2, 0.3, 0.4, 0.5, 0.6, 0.9, 1.0, 1.6, and 2.0 mm. A set of parameters, denoted as the ‘Carr indices’, was determined using a Hosokawa Powder Tester (Hosokawa Micron B.V., Doetinchem, The Netherlands) following the methods recommended by the corresponding ASTM standard [25]. The poured density *ρ*_0_ and tapped density ρ_1_ of the materials were determined on a Hosokawa Powder Tester (Hosokawa Micron B.V., Doetinchem, The Netherlands), Type PT-S [26]. The poured density ρ_0_ (in kg/m^3^) represents the bulk density of loose particulate deposits built up in a vessel by dropping according to the injection method. The packed density ρ_1_ (in kg/m^3^) represents the bulk density of the tapped loose material. The tapped density was obtained by tapping the sample 180 times. The popular Carr compressibility index (CI%) [27] was also determined using the PT-S tester, so as to describe the flowability. It was calculated as follows: CI(%) = 100 × (ρ_1_–ρ_0_)/ρ_1_. A higher compressibility is associated with a lower flowability. In industrial practice, it is assumed that powder flows easily when the CI is in the range of 5% to 15%. The bulk density and tapped density were also determined using the PT-S tester. Another popular parameter, the Hausner ratio (HR), defined as HR = ρ_1_/ρ_0_, could also be calculated using this tester. In industrial practices, it is considered that durable pellets can be produced from powders with HR values no higher than 1.6.

Raw materials with moisture contents of 8% and 20% were used to produce the pellets. Distilled water was added, and the materials were mixed in a laboratory mixer for 15 min per h within 24 h to achieve 20% moisture content. The moisture content levels corresponded to those existing in technological practice for granulate production. Castellano et al. [24] conducted experiments on materials with moisture contents between 10% and 12%. As reported by Kaliyan and Morey [6], such moisture levels are commonly used in granule production. Samples of 200–300 g were weighed before and after 24 h of drying at 105 °C in a laboratory furnace to estimate the moisture content. The moisture content was calculated ‘wet’. The moisture content values were within the range recommended by Kaliyan and Morey [6], Russel et al. [7] and Lu and Liu [8]. Kaliyan and Morey [6] suggested that at a 20% moisture content, free water formation and weakening of the bonds occurs between the individual particles of the sawdust. For this reason pellets at higher moisture contents were not tested. Oak and pine biomasses with a moisture content of approximately 8% were used by Lu and Liu [8] and they concluded that high-quality pellets could be produced when the initial moisture content of the materials ranged from 6% to 12%. In our experiments we extended the m.c. range for exploratory purposes. This extension of range of tested m.c. was suggested by wood industry experts.

**Figure 1:**
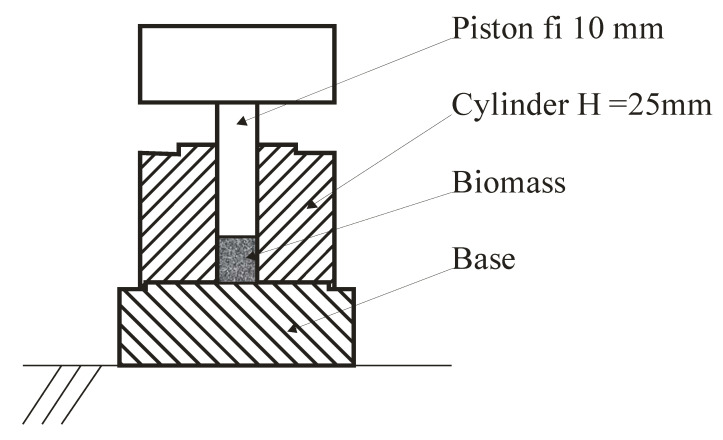
Test pelletizer used for biomass compression.

The materials were compacted in a cylindrical die with a diameter of 10 mm and height of 25 mm. Figure 1 shows the arrangement of the experimental pelletiser. A cylindrical die was placed on a steel table and filled with a loose material. The sample filled the entire volume of the cylinder. After filling, the piston was placed on the surface of the material, and compaction was performed using a high-pressure actuator and yoke. Two compaction pressure values were applied in the experiments: 60 and 120 MPa. The compaction pressure was measured using a load cell mounted between the piston and yoke. The compaction pressure was within the range recommended by other researchers [8]. A review of the literature has shown that the compaction pressure should be in the range of 100–150 MPa. In this study, compaction pressures ranging from 60 MPa to 120 MPa were applied, following Li and Liu [8]. The compacted pellets were removed from the cylinder with the same piston after changing the base for the second one, which had a hole 1 mm larger than the cylinder diameter.

A drop test was conducted through the free fall of a single pellet from a height of 1 m onto a concrete surface. Ten pellets, each approximately 10 mm long, were tested for each variant of the experiment. The mass of each pellet was measured before and after dropping. The percentage of the weight loss was used as a measure of the durability.

The strength of the pellets was tested by means of cross-compression between a round plate and stamp, as commonly used for pharmaceutical tablets [28]. The stamp was moved down at a constant rate of deformation of 0.033 mm/s. The compressive force (in N) and the displacement of the moving stamp (in mm) were recorded in real time. The maximum compression force at the breakage point B was measured, and the breakage strength of the agglomerates σ_B_ was calculated following Fell and Newton [29]: $\sigma_{B}=\frac{2F_{B}}{\pi \mathrm{dh}}$, where: F_B_—breakage force, d—tablet diameter, and h—tablet height. The tests were replicated 10 times.

The heat of combustion was determined using a calorimetric method according to the ISO standard [30] using a semi-automatic AC 600 device (LECO Corporation, St. Joseph, MI, USA). The tablets produced from the tested material, weighing 1 g, were burned in a calorimetric bomb in an oxygen atmosphere of 3 MPa. Ignition was initiated using a cantaloupe resistance wire. The heat of combustion was determined using the ‘TrueSpeed’ method, and was calculated automatically using the AcWin software (1.12, LECO Corporation, St. Joseph, MI, USA). The combustion heat yielded a lower calorific value when subtracting the evaporation heat. The ash content was determined according to the corresponding ISO standard [31]. The measurements were performed in three repetitions.

Analysis of the variance of the obtained data was performed using the STATISTICA (12 Package; StatSoft, Tulsa, OK, USA) [32]. The information on the statistical significance was given as follows: the 0.95 confidence intervals are shown as bars in the figures, p denotes the probability (or statistical significance level), and F is the value of the Snedecor test function. The higher the F value, the stronger the influence of a given factor on a given parameter.

## 3. Results

The size distributions of the sawdust particles are presented in Table 1. Although the sawdust was obtained using the same cutting tool for different types of wood, the sieve analysis showed that the contents of the individual fractions were different.

For pine sawdust, the highest content of 28.8% was obtained for the particles that were not deposited on the sieve, i.e., 0.1 < d < 0.2. The lowest percentage was approximately 0.1% is obtained for the fractions 1.0 < d < 1.6, d > 2.0, and 1.6 < d < 2.0. In the case of pine sawdust, the content of the remaining fractions ranged from 3.9% for the fraction 0.9 < d < 1.0 to 15.2% for the fraction 0.3 < d < 0.4. In the case of birch sawdust, the highest content of 23.7% was obtained for the fraction 0.3 < d < 0.4. The lowest percentages (below 0.4%) was obtained for the largest fractions, above d > 1.0. The contents of 2.6% and 22.3% were obtained for the 0.9 < d < 1.0 and 0.1 < d < 0.2 fractions, respectively. For oak sawdust, a fraction content below 0.6% was obtained for the fraction 1.0. The highest n contents, from 13.9% to 23.3%, were obtained for the fraction d < 0.4. For the 0.4 < d < 1.0 fraction, the content ranged from 6.8% to 9.7%.

For poplar sawdust, similar to the cases with the other materials, the contents of the largest fractions were the smallest above 1.0 and are below 0.3%. For poplar sawdust, the highest content of 25.4% was obtained for the 0.9 < d < 1.0 fraction, and the lowest content of 4.8% was obtained for the 0.6 < d < 0.9 fraction. For willow sawdust, the highest content of 17.6% was obtained for the 0.5 < d < 0.6 fraction. The content of fractions above 1.0% was less than 1%. For the remaining fractions, their contents ranged from 7.7% for the d < 0.1 fraction to 15.1% for the 0.1 < d < 0.2 fraction. In the case of sawdust beech wood, the highest content of 16% was obtained for the fraction 0.1 < d < 0.2. The lowest content was obtained for the fraction with d > 1.0. Harder deciduous tree materials such as birch, oak, and beech have a higher content of finer particles (less than 0.1). A high fraction content of 0.9 < d < 1.0 was characteristic of a case of chopped-up, fibrous poplar wood material.

Table 2 presents the poured and tapped densities, and the parameters describing the flowability of the granular material: the CI (%) and HR. The density of the pine sawdust with 8% moisture content was 144 kg/m^3^, whereas for 20% moisture content was 130 kg/m^3^. For birch sawdust with 8% moisture content, the poured density was 195 kg/m^3^, and decreased to 188 kg/m^3^ with increasing moisture content.

The highest poured density of 248 kg/m^3^ was obtained for oak sawdust. An increase in the moisture content of the material to 20% resulted in a decrease in the poured density to 204 kg/m^3^. The lowest poured densities were obtained for poplar sawdust and beech sawdust. For a moisture content of 8%, the poured density was 136 kg/m^3^, and for a moisture content of 20%, was 122 kg/m^3^ and 120 kg/m^3^, respectively. The density of sawdust obtained from willow wood with a moisture content of 8% was 156 kg/m^3^, and decreased to 130 kg/m^3^ with an increase in the moisture content.

The highest differences between the poured and tapped densities were observed for pine sawdust. After tapping, the density of the material increased by 40% and 42% for 8% and 20% moisture contents, respectively. For oak and birch sawdusts with 8% moisture content, the increases in density after tapping were 24% and 36%, respectively. The highest increase in density was observed for birch sawdust with 20% moisture content. In this case, the tapped density was 172 kg/m^3^. The lowest increase in the density of the material after tapping (29%) was observed for oak sawdust. The values of the poured and tapped densities of polydisperse blends were strongly affected by the shapes and surface conditions of the single particles. This effect was stronger in materials with a higher moisture content (Stasiak et al., [13]). The slight differences in the tapped densities of materials composed of different numbers of small and large particles could be owing to the relatively rigid structures produced by large particles, with the small particles moving freely and filling the voids between larger elements.

The analysis of the HR and compressibility (1.24 < HR < 1.43; CI = 22–30%) indicated that, regardless of the moisture content of the sawdust, the examined materials were cohesive, and have passable or poor flow properties. The oak sawdust with a moisture content of 8% was characterized by the highest flowability. In this case, HR = 1.24 and CI = 19%. From the point of view of pharmaceutical technology, materials with HR values less than 1.6 may produce durable tablets. This condition was fulfilled by all tested materials.

As noted above, the pellets were produced from each type of wood sawdust in a laboratory pelletiser. They were taken out directly from the pelletiser after compaction of the material under loads of 60 and 120 MPa. Pellets with regular cylindrical shapes were obtained. The pellets compacted with smaller loads were higher than those compacted with larger loads (Table 3).

The surfaces of the pellets made of oak and beech sawdust at the compaction load of 120 MPa were darkened as a result of, e.g., the temperature increase in the cylinder during the compaction process. This can also be caused by the hardness values of the individual particles of the oak and beech sawdusts, which were higher than those of other materials. In the case of oak, the intensity of the colour was also owing to the high tannin content in the wood. Discolouration was less visible in pellets made of beech sawdust.

**Figure 2:**
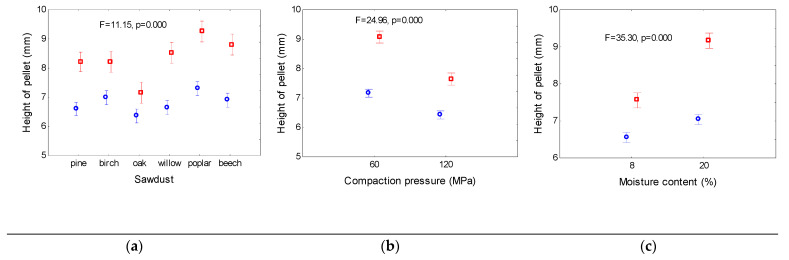
Mean values of pellet height after realized (blue circles) from die and after 2 h (red squares) as dependent on sawdust origin (**a**), compaction pressure (**b**) and moisture content (**c**). Mean values in columns are calculated for whole range of parameters from two other. Vertical bars denote 0.95 confidence intervals; F, p—analysis of variance parameters.

The height of the pellets was measured directly after the pelleting process and after conditioning under laboratory conditions for 2 h. Figure 2 shows that directly after compaction, the height of the pellets made of oak sawdust was the smallest, at 6.5 mm. The pellets with the greatest height of 7.3 mm were made of poplar sawdust. For pine, birch, willow, and beech sawdust, the height ranged from 6.5 to 7.0 mm.

After conditioning under laboratory conditions, the height of the pellets increased. The highest increases in height were found for willow, poplar, and beech (28%, 26%, and 27%, respectively). The smallest increase in height (12%) was observed for the oak pellets. For pellets made of pine and birch sawdust, the increases in height were 27% and 17%, respectively. The differences between the heights of pellets made of various materials were owing to the differences in the hardness of the individual sawdust particles. The poplar and willow particles were the most elastic. Therefore, after 2 h of conditioning, the largest increases were observed in the heights of the pellets composed of these materials. The sieve analysis conducted for birch and oak shows the highest proportion of finest particles (below 0.1 mm) in these materials. The smallest particles may block the larger particles, resulted in the smallest increase in the height of the pellets after 2 h of conditioning. An increase in the compaction pressure resulted in a decrease in the height of the pellets. The average heights immediately after the pelleting process were approximately 7.25 mm and 6.3 mm for compaction pressures of 60 MPa and 120 MPa, respectively. For a higher compaction pressure, the increases in the heights of the pellets after 2 h of conditioning were smaller, i.e., 27% and 18% for compaction pressures of 60 MPa and 120 MPa, respectively. An increase in the moisture content of the sawdust resulted in higher heights of the pellets, and a stronger tendency to grow after 2 h of storage in the laboratory. This is owing to an increase in the elasticity of the individual granules and elastic deformation range with an increase in the moisture content. The average height of pellets made of sawdust with 8% moisture content was 6.6 mm after the pelleting process, and approximately 7.6 mm after 2 h of conditioning. For sawdust with 20% moisture content, the height of the pellets was approximately 7 mm after pelleting and approximately 9.25 mm after 2 h.

**Figure 3:**
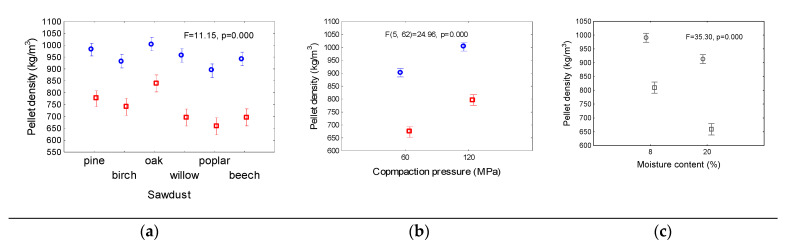
Mean densities of pellets after realized (blue circles) from die and after 2h (red squares) as dependent on sawdust origin (**a**) compaction pressure (**b**) and moisture content (**c**). Mean values in columns are calculated for whole range of parameters from two other. Vertical bars denote 0.95 confidence intervals; F, p—analysis of variance parameters.

The mean densities of the pellets were shown in Figure 3. The largest mean density of the pellets was obtained for oak sawdust. The densities were approximately 1000 kg/m^3^ and 840 kg/m^3^ after the pelleting process and after conditioning in the laboratory, respectively.

The smallest mean densities of 892 kg/m^3^ and 658 kg/m^3^ were obtained for the poplar pellets. For the other materials, the density of pellets directly after compression ranged from 932 kg/m^3^ (for birch) to 981 kg/m^3^ (for pine). After 2 h of conditioning, the density ranged from 696 kg/m^3^ (poplar) to 774 kg/m^3^ (pine). The mean densities calculated for all the materials and parameter variables were dependent on the compaction pressure, and are smaller for smaller compaction pressures.

After 2 h of conditioning, the mean densities of pellets compressed at a pressure of 60 MPa decrease by 25%, from approximately 900 kg/m^3^ to approximately 675 kg/m^3^. For higher consolidation pressures, the density decreased by approximately 20%. This is a result of the stronger bonds between the particles compacted at higher pressures. The density of the pellets was based on the moisture content of the material. An increase in the moisture content from 8% to 20% resulted in a nearly 10% decrease in the mean density of the pellets removed from the punch. In the case of pellets stored in the laboratory for 2 h, the density decreased by approximately 20%. An increase in moisture content resulted in a higher flexibility of the single particles of the wood sawdust. Free water can appear on the surface of the particles, facilitating stress relaxation and loosening of the pellet structure.

**Figure 4:**
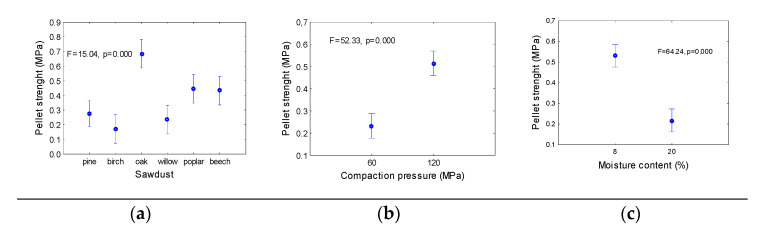
Mean values of strength of the pellets as dependent on sawdust origin (**a**), compaction pressure (**b**) and moisture content of sawdust (**c**). Mean values in columns are calculated for whole range of parameters from two others. Vertical bars denote 0.95 confidence intervals; F, p—analysis of variance parameters.

**Figure 5:**
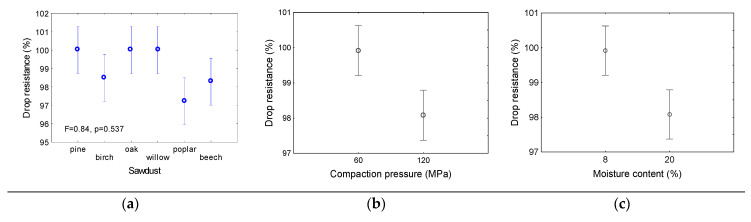
Mean values of drop resistance as dependent on sawdust origin (**a**) compaction pressure (**b**) and moisture content (**c**). Mean values in columns are calculated for whole range of parameters from two others. Vertical bars denote 0.95 confidence intervals; F, p—analysis of variance parameters.

Figure 4 and Figure 5 show the results obtained from the standard pellet strength tests. Figure 4 showed the mean strengths of the pellets, as determined by pressing a pellet between two parallel planes. The highest strength (approximately 0.7 MPa) was obtained for oak pellets, and the lowest strength (approximately 0.18 MPa) was obtained for birch sawdust.

The strengths of pellets made of pine and willow sawdust were similar, ranging between 0.25 and 0.28 MPa. For poplar and beech pellets, the strengths were higher, i.e., 0.45 MPa. The strength of the pellets was strongly determined by the compaction pressure. An increase in the compaction pressure from 60 MPa to 120 MPa resulted in an increase in the pellet strength by more than 100%, from approximately 0.25 MPa to 0.5 MPa. An opposite effect was observed for the moisture content of the sawdust. With an increase in the moisture content of material, the average strength of the pellets decreased by more than half, from 0.55 MPa to approximately 0.2 MPa. The second strength pellet parameter analysed in this study was the drop resistance. This parameter showed relatively high values for all of the examined pellets.

The smallest drop resistance was obtained for poplar pellets, and is approximately 97%. A slightly higher value of approximately 98% was obtained for birch and beech. A 100% drop resistance was obtained for pellets made of pine, oak, and willow.

A decrease in the drop resistance was observed with an increase in the compaction pressure. This effect was opposite to that observed for the crush resistance, and was probably owing to the dynamic loading of a single pellet during dropping. A decrease in the drop resistance was also observed with an increasing moisture content of the sawdust. The results showed that the type of dynamic or static loading determined the strength of the pellets. For all of the tested materials, the drop resistance remained at a high level, and was acceptable for industrial practice.

The results of the combustion tests are listed in Table 4. The heat of combustion for pellets made of sawdust with higher moisture contents were larger. This indicates that there was water left over from the pellet-making process which does not evaporate, and that part of the heat was used for the evaporation of the water. The highest heat of combustion was obtained for pine pellets (18.97 and 16.43 at moisture contents of 8 and 20%, respectively). For the other pellets, the values of the parameter were approximately 17.5 MJ/kg and 15.45 MJ/kg at moisture contents of 8 and 20%, respectively.

Significant differences in the ash content in dry mass are observed for the various sawdust samples. The ash content is the highest for willow pellets, at 1.52%. The smallest ash content (0.32%) was obtained from the pine pellets. A high ash content of 0.94% was observed for the poplar pellets.

## 4. Discussion

The results presented herein provide useful information for producers and users of biomass. In this study, wood species commonly used in Eastern Europe were examined, providing technological information which can help producers of pellets to maintain high-quality products. The punch-and-die process was conducted following Li and Liu [8]; however, in this study, pellets with smaller diameters were produced. It was observed that, at room temperature, sawdust could be quickly compacted into pellets of good quality. The findings corroborate those reported by Li and Liu, in spite of using an agglomeration device with an almost fivefold smaller diameter and a much smaller height. The experimental pelletiser applied in this study was also smaller than that used by Krizan et al. [17], who produced pellets in a pelletiser with a diameter of 20 mm.

In this study, pellets were produced from the starting materials with moisture contents of 8% and 20%. The moisture content values were within the range recommended by Kaliyan and Morey [6], Russel et al. [7] and Lu anf Liu [8] as the most appropriate for producing pellets of good quality. Kaliyan & Morey [6] suggested that at a 20% moisture content, free water formation and weakening of the bonds occurs between the individual particles of the sawdust. This results in the omission of pellets at higher moisture contents. Oak and pine biomasses with a moisture content of approximately 8 % were used by Lu and Liu [8]. These authors concluded that high-quality pellets could be produced when the initial moisture content of the materials ranged from 6% to 12%. They recommended a moisture content of 8% as the most appropriate for the production of pellets. The results of the present study showed that this range may be extended while maintaining a high level of pellet quality. The range of moisture content in the starting materials for pellet production was the same as that used by Russel et al. [7]. The compaction pressure applied in the present study was also within the range recommended by other researchers [8]. A review of the literature has shown that the compaction pressure should be in the range of 100–150 MPa. In this study, compaction pressures ranging from 60 MPa to 120 MPa were applied, following Li & Liu [8].

The pellets produced for the purpose of this project had a density approximately 30% higher than those obtained for agricultural biomass by Niedziółka et al. [10]. Adapa et al. [15] presented the compaction characteristics of four types of straw. The densities of wheat pellets produced by these authors ranged from approximately 850 kg/m^3^ to 924 kg/m^3^, corresponding to the densities determined in the present study. The densities of oak and pine sawdust were comparable with those obtained by Li and Liu [8]. In the present study, the density of the pellets decreased with increasing moisture content, and increased with increasing compaction pressure. Li and Liu [8] observed the same effects for logs made of oak and pine sawdust.

The results of the present study are comparable with those obtained for beech sawdust by Krizan et al. [17]. These authors observed similar decreases in the density of pellets after their conditioning. They also reported decreases in the height and density of pellets with an increase in compaction pressure.

The determined parameters of pellets made from different woods show that the fibrous biomass and interlocking mechanism at the mechanical level are the main causes of the binding mechanism in densified products. The density of the pellets depended strongly on the moisture content of the feedstock, and decreased with an increase in the storage time. Gilbert et al. [5] observed a negative influence on the density of switch grass from the moisture content and storage time. The present study has shown the detrimental effects of larger particles and fibrous particles on the deterioration of agglomerates, thereby agreeing with the findings reported by Li and Liu [8].

The pellet strength tests simulated the loads subjected to pellets during storage in silos [6]. The pellet strength determined in this study did not exceed 1 MPa, i.e., much smaller than those obtained by Graham et al. [12]. These authors found that the shear modulus ranged from 5 to 250 MPa. This significant difference is a result of the three-point fracture test and the different stress states in the specimen under loading. An identical strength test was presented by Russel et al. [7] in their study modelling the deformations of pellets under loads.

The durability of the examined pellets corresponds to the durability of the corresponding agglomerate, as reported by the standards and by Jarvinen and Agar [2]. The durability values of the pellets in this study are higher than those obtained for industrial pellets stored in large quantities and examined by, inter alia, Graham et al. [1]. This difference indicates the strong influence of weather conditions and storage time on pellet durability. The results presented in this study are comparable to those obtained by Li and Liu [8] for compacting pine and oak sawdust under compaction pressures ranging from 34 to 138 MPa. However, the values of the compression strengths determined for the pallets examined in the present study were significantly smaller than those obtained by Li and Liu [8].

The impact resistance test conducted for this project simulated conditions prevailing during the discharge of pellets from bins or trucks. The results are comparable to those reported by other researchers [8].

The calorific values determined for the examined pellets and ash content were comparable to those obtained by Graham et al. [1] for steam-exploded and white wood pellets. A slight decrease in the calorific value was observed with an increase in the moisture content of the material. The calorific values for beech and pine sawdust were similar to those determined by Jarvinen and Agar [2]. However, in the present study, smaller calorific values were obtained for pine pellets, and larger calorific values were obtained for beech pellets. These differences indicate that the presence of resin in the wood increases its calorific value. Duca et al. [9] concluded that ash content could be a good parameter for rapid quality assessments. They found that the ash content was significantly lower in coniferous pellets than in broadleaf pellets. This finding was not confirmed in the present study. The quality parameters were the lowest for willow and poplar pellets.

## 5. Conclusions

The properties of the pellets were determined in terms of the type of wood and the granulometric distribution of sawdust particles. Hard wood types have a higher content of fine fractions than flexible and fibrous wood.

The density of sawdust made of hardwood increased after tapping. From the point of view of pharmaceutical technology, materials with HR values not higher than 1.6 can produce durable tablets. This condition was fulfilled for all of the examined materials.

The pellets made of different types of sawdust had various heights. In some cases, the surfaces of pellets were browned owing to an increase in temperature, as a result of higher friction. The height of the pellets increased after 2 h of laboratory conditioning. The largest increase in height was obtained for willow, poplar, and beech pellets, whereas the smallest increase was observed for oak pellets. An increase in the pressure of the compaction resulted in more compacted, more stable pellets with a lower 2 h expansion. An increase in the moisture content of the sawdust resulted in higher heights of the pellets, and a stronger tendency to expand.

The largest mean density of pellets was obtained for the oak sawdust pellets. The smallest mean density was obtained for the poplar pellets. The average density calculated for all of the materials and parameter variables was dependent on the compaction pressure. It was smaller for a compaction pressure of 60 MPa, and larger for a compaction pressure of 120 MPa. The average densities also decreased after 2 h of storage under laboratory conditions.

The highest strength was obtained for oak pellets, and the lowest for birch sawdust. An increase in the compaction pressure from 60 MPa to 120 MPa resulted in an increase in the strength of the pellets by more than 100%. The average strength decreased by 65% with an increase in the moisture content.

For all of the tested materials, the drop resistance remained at a high level, and was acceptable for industrial practice. The lowest value of drop resistance was obtained for poplar pellets. An increase in the compaction pressure resulted in a decrease in the drop resistance. The same effect was observed for the moisture content.

The highest calorific values were obtained for pine pellets. The heat of combustion was smaller for pellets made of sawdusts with higher moisture contents.

The highest ash value was obtained for willow pellets, and the lowest value was obtained for pine pellets. A high ash content was observed for poplar pellets.

## Figures and Tables

**Table 1:** Size distribution of different sawdust biomass materials with moisture content of 8%.

Particle Size (mm)	Pine Fraction (%)	Birch Fraction (%)	Oak Fraction (%)	Poplar Fraction (%)	Willow Fraction (%)	Beech Fraction (%)
<0.1	9.2	12.7	13.1	5.3	7.7	10.4
0.1–0.2	28.8	22.3	23.3	13.0	15.1	16.0
0.2–0.3	14.6	15.2	13.9	8.8	10.3	9.8
0.3–0.4	15.2	23.7	15.9	15.3	16.9	14.0
0.4–0.5	12.1	10.2	9.7	5.4	9.3	8.2
0.5–0.6	7.8	8.6	7.7	21.5	17.6	12.9
0.6–0.9	8.0	4.0	7.8	4.8	9.1	14.1
0.9–1.0	3.9	2.6	6.8	25.4	12.3	13.8
1.0–1.6	0.1	0.2	0.3	0.2	0.3	0.2
1.6–2.0	0.1	0.3	0.6	0.3	0.9	0.6
2.0>	0.1	0.1	0.6	0.2	0.9	0.5

**Table 2:** Bulk and tapped densities of experimental materials.

Material	Moisture Content m.c. (%)	Bulk Density ρ_B_ (kg/m^3^)	Tapped Density ρ_T_ (kg/m^3^)	Compressibility CI (%)	Housner Ratio HR
Pine	8	144 ± 2	202 ± 2	28.7 ± 1.5	1.40
	20	131 ± 1	185 ± 0	29.3 ± 0.9	1.42
Birch	8	195 ± 1	267 ± 2	27.0 ± 0.9	1.36
	20	188 ± 4	246 ± 1	23.3 ± 1.6	1.30
Oak	8	248 ± 5	308 ± 0	19.5 ± 1.7	1.24
	20	205 ± 1	264 ± 2	22.6 ± 0.9	1.29
Poplar	8	137 ± 1	178 ± 4	23.2 ± 1.3	1.30
	20	122 ± 2	162 ± 0	24.7 ± 1.2	1.32
Willow	8	156 ± 1	200 ± 4	22.0 ± 1.0	1.28
	20	131 ± 3	175 ± 3	25.5 ± 1.9	1.34
Beech	8	136 ± 2	176 ± 2	22.5 ± 1.5	1.29
	20	120 ± 4	172 ± 3	30.1 ± 3.3	1.43

**Table 3:** Typical images of produced pellets at 60 and 120MPa of compaction pressure.

Pine	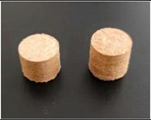
Birch	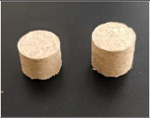
Oak	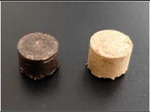
Poplar	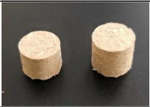
Willow	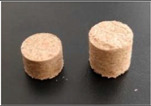
Beech	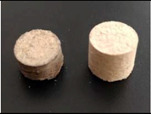

**Table 4:** Heat of combustion HC, of tested sawdust.

Wood Sawdust	Moisture Content (%)	Heat of Combustion (LHV) (MJ/kg)	Ash Content in Dry Mass (%)
pine	8	18.97 ± 0.03	0.32
	20	16.43 ± 0.10	
birch	8	17.54 ± 0.12	0.36
	20	15.58 ± 0.09	
oak	8	17.52 ± 0.06	0.41
	20	15.44 ± 0.04	
poplar	8	17.48 ± 0.04	0.94
	20	15.43 ± 0.76	
willow	8	17.72 ± 0.02	1.52
	20	15.58 ± 0.02	
beech	8	17.29 ± 0.09	0.41
	20	15.40 ± 0.07	

## Data Availability

Data available on request.
